# 467. Maternal Antibody and Placental Transfer Increases with Hybrid Immunity Against SARS-CoV-2 but Transferred Antibody is Qualitatively Different

**DOI:** 10.1093/ofid/ofad500.537

**Published:** 2023-11-27

**Authors:** Ketrah Dekanich, Kristen Smith, Eric D Laing, Wei Wang, Carol D Weiss, Emily C Samuels, Isabella Swafford, Dominic Paquin-Proulx, Jeffrey R Currier, Emily Parsons, Stephanie A Richard, Milissa U Jones, Julia Rozman, Rhonda E Colombo, Nikhil Huprikar, David Lindholm, Katrin Mende, Anuradha Ganesan, Derek Larson, Evan Ewers, Ryan Flanagan, Mark P Simons, Robert O’Connell, David Tribble, Brian Agan, Timothy Burgess, Patrick W Hickey, Simon Pollett, Allison M Malloy

**Affiliations:** Uniformed Services University, Bethesda, Maryland; Walter Reed National Military Medical Center, Bethesda, Maryland; Department of Microbiology and Immunology, Uniformed Services University, Bethesda, MD, Bethesda, Maryland; US Food and Drug Administration, Silver Spring, Maryland; U.S. Food and Drug Administration, Silver Spring, Maryland; HJF, USUHS, Bethesda, Maryland; Walter Reed Army Institute of Research, Bethesda, Maryland; Walter Reed Army Institute of Research, Bethesda, Maryland; Walter Reed Army Institute of Research, Bethesda, Maryland; Uniformed Services University, Bethesda, Maryland; Infectious Disease Clinical Research Program, Department of Preventive Medicine and Biostatistics, Uniformed Services University of the Health Sciences, Bethesda, MD, USA, Bethesda, Maryland; Uniformed Services University, Bethesda, Maryland; Infectious Disease Clinical Research Program, USUHS, Bethesda, Maryland; Infectious Disease Clinical Research Program, USUHS; Henry M. Jackson Foundation for the Advancement of Military Medicine, Inc., Bethesda, Maryland; Walter Reed National Military Medical Center, Bethesda, Maryland; Department of Medicine, Uniformed Services University of the Health Sciences; Brooke Army Medical Center, San Antonio, Texas; 1Infectious Disease Clinical Research Program, Department of Preventive Medicine and Biostatistics, Uniformed Services University of the Health Sciences and Brooke Army Medical Center, JBSA Fort Sam Houston, TX, San Antonio, TX; Infectious Disease Clinical Research Program, USUHS; Henry M. Jackson Foundation for the Advancement of Military Medicine Inc, Bethesda, Maryland; Fort Belvoir Community Hospital and Uniformed Services University, Fort Belvoir, Virginia; Fort Belvoir Community Hospital, Fort Belvoir, Virginia; Tripler Army Medical Center, Honolulu, Hawaii; Infectious Disease Clinical Research Program, Department of Preventive Medicine and Biostatistics, Uniformed Services University of the Health Sciences, Bethesda, MD, USA, Bethesda, Maryland; Infectious Disease Clinical Research Program, USUHS, Bethesda, Maryland; Infectious Disease Clinical Research Program, Department of Preventive Medicine and Biostatistics, Uniformed Services University of the Health Sciences, Bethesda, MD, USA, Bethesda, Maryland; Infectious Disease Clinical Research Program, Department of Preventive Medicine and Biostatistics, Uniformed Services University of the Health Sciences, Bethesda, MD, USA, Bethesda, Maryland; Infectious Disease Clinical Research Program, Department of Preventive Medicine and Biostatistics, Uniformed Services University of the Health Sciences, Bethesda, MD, USA, Bethesda, Maryland; Uniformed Services University, Bethesda, Maryland; Infectious Disease Clinical Research Program, Department of Preventive Medicine and Biostatistics, Uniformed Services University of the Health Sciences, Bethesda, MD, USA, Bethesda, Maryland; Department of Pediatrics, Uniformed Services University of the Health Sciences, Bethesda, MD, USA, Bethesda, Maryland

## Abstract

**Background:**

Defining the magnitude and quality of antibodies induced by infection and vaccination for SARS-CoV-2 during pregnancy and that cross the placenta can improve strategies to protect both mothers and infants.

**Methods:**

Maternal peripheral and neonatal cord blood were collected from participants enrolled in a prospective longitudinal cohort study, the Epidemiology, Immunology, and Clinical Characteristics of pathogens with pandemic potential (EPICC), after presenting to a military treatment facility between March 2020 and March of 2022 with either COVID-19 or receipt of the COVID-19 vaccine. SARS-CoV-2 spike-specific IgG and neutralizing titers (NT) were measured with a multiplex microsphere immunoassay and a pseudovirus assay, respectively. Antibody dependent cellular cytotoxicity (ADCC), antibody dependent cellular phagocytosis (ADCP) and opsonization were measured with cell and bead-based assays.

**Results:**

During the study period 20 pregnant participants were enrolled. Paired biospecimens (maternal blood/cord blood) were collected near birth from 17; 11 had a history of SARS-CoV-2 infection and 6 had both infection and received the BNT162b2 vaccine during pregnancy. The average anti-spike IgG at time of birth from mothers who were infected versus infected and vaccinated during pregnancy was 165.5 and 2592.7 BAU/ml, respectively (p=0.006). Average NT titers in infected versus infected and vaccinated mothers were 131.7 and 971.5 IC50, respectively (p = 0.0001). Anti-spike IgG and NT were not significantly different within dyads. Interestingly, ADCC was generally higher in newborn cord blood compared to maternal peripheral blood by 2.5-fold in both groups, while ADCP and opsonization was not.

**Conclusion:**

Our study showed that hybrid immunity, comprised of infection and vaccination during pregnancy, increased the total magnitude and neutralizing potential of anti-SARS-CoV-2 antibodies in mothers and infants. Both hybrid immunity and isolated infection resulted in an increased transfer of ADCC activity across the placenta. Hybrid immunity is now common and determining the effect on maternal antibody levels and the characteristics of those antibodies can help inform maternal immunization strategies and protocols.

**Disclosures:**

**Julia Rozman, BS**, AstraZeneca: TBD **Mark P. Simons, PhD**, AstraZeneca: The IDCRP and HJF were funded to conduct an unrelated phase III COVID-19 monoclonal antibody immunoprophylaxis trial as part of US Govt COVID Response **David Tribble, MD, DrPH**, AstraZeneca: The IDCRP and HJF were funded to conduct an unrelated phase III COVID-19 monoclonal antibody immunoprophylaxis trial as part of US Govt COVID response **Timothy Burgess, MD, MPH**, AstraZeneca: The IDCRP and the Henry M. Jackson Foundation (HJF) were funded to conduct an unrelated phase III COVID-19 monoclonal antibody immunoprophylaxis trial **Simon Pollett, MBBS**, AstraZeneca: The IDCRP and the Henry M. Jackson Foundation (HJF) were funded to conduct an unrelated phase III COVID-19 monoclonal antibody immunoprophylaxis trial

